# Illuminating the physiology of extracellular vesicles

**DOI:** 10.1186/s13287-016-0316-1

**Published:** 2016-04-16

**Authors:** Hongyoon Choi, Dong Soo Lee

**Affiliations:** Department of Nuclear Medicine, Seoul National University College of Medicine, Seoul National University Hospital, 28 Yongon-Dong, Jongno-Gu, Seoul 110-744 South Korea; Department of Molecular Medicine and Biopharmaceutical Sciences, Graduate School of Convergence Science and Technology, Seoul National University, Gwanak _599 Gwanak-ro, Gwanak-gu, Seoul 151-742 South Korea; College of Medicine or College of Pharmacy, Seoul National University, Gwanak _599 Gwanak-ro, Gwanak-gu, Seoul 151-742 South Korea

**Keywords:** Extracellular vesicle, Exosome, Microvesicle, Imaging, In vivo distribution

## Abstract

Extracellular vesicles play a crucial role in intercellular communication by transmitting biological materials from donor cells to recipient cells. They have pathophysiologic roles in cancer metastasis, neurodegenerative diseases, and inflammation. Extracellular vesicles also show promise as emerging therapeutics, with understanding of their physiology including targeting, distribution, and clearance therefore becoming an important issue. Here, we review recent advances in methods for tracking and imaging extracellular vesicles in vivo and critically discuss their systemic distribution, targeting, and kinetics based on up-to-date evidence in the literature.

## Background

Extracellular vesicles (EVs) released from a variety of cells target recipient cells for intercellular communication and transfer a subset of genetic materials and proteins [1–3]. Until now, diverse roles have been identified for EVs, ranging from immune modulation [4–6], to neuron–glial communication [7, 8], to stem cell-injured tissue interaction [9], to the pathophysiological processes of cancer metastasis [10–12].

EVs include a broad spectrum of vesicles secreted by several types of cells and the term is used as a collective one. These include exosomes, ectosomes, oncosomes, shed vesicles, and microvesicles. Thus, EVs represent a broad spectrum of vesicles secreted by several types of cells. Among these, exosomes are small (30–100 nm) vesicles derived from the endosomal pathway while microvesicles are of various sizes and generated by budding of the plasma membrane (50–1000 nm) [3, 5]. However, discriminating exosomes from microvesicles is difficult based on their physical properties or protein composition. Because of this overlap, we discuss imaging results and physiology of EVs referring to both types of EVs.

Genetic materials or proteins conveyed by EVs functionally change the recipient cells [13], and therefore visualization and tracking of EVs currently receive great attention as a way to reveal their physiology. Recently developed imaging techniques provide direct evidence of cellular phenotypic change as a consequence of intercellular transfer of genetic materials mediated by EVs. Tracking of exogenous engineered EVs also became an important issue for their diagnostic and therapeutic use [14–16] in cancer [17, 18] and in regenerative medicine [19]. EVs can transport genetic materials and proteins, and thus have potential as a drug carrier [17, 20, 21]. Despite the high expectation of therapeutic potential, the lack of knowledge of in vivo behavior of EVs is a major drawback. Noninvasive imaging enables us to understand the in vivo distribution and the fate of EVs and to elucidate their targeting capability, and here we review these imaging approaches and critically discuss the physiology of EVs revealed by the imaging and tracking studies.

## Tracking methods for extracellular vesicles

### Fluorescence labeling

**Fig. 1:**
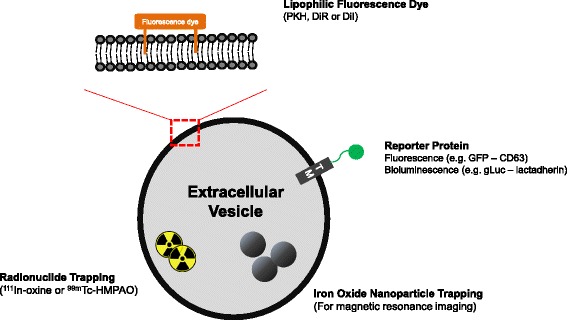
Strategy for visualization of EVs. The size of EVs is around 100 nm, which restricts direct imaging with optical microscopy. Recently, several labeling methods have been developed. Lipophilic fluorescence dye is simple and commonly used to track EVs. Reporter imaging using fluorescence or bioluminescence combined with transmembrane proteins could provide information more specific to EVs than direct dye labeling. For clinical application and deep tissue imaging, radionuclide imaging or MRI could be possible using ^111^In-oxine, ^99m^Tc-HMPAO, and iron oxide nanoparticles. *GFP* green fluorescent protein

The imaging and tracking strategy for EVs is summarized in Fig. 1. Direct fluorescence labeling of EVs has been widely used to investigate in vivo behavior of exogenous EVs. Fluorescence labeling could provide whole-body images on highly sensitive optical cameras as well as fluorescence microscopic images. Thus, EVs labeled with dyes can be widely used for microscopic identification of EVs to reveal intercellular communication and for tracking systemically administered EVs. Lipophilic dyes including PKH, DiI, and DiR are commonly used and yield stable fluorescent signals [11, 18, 22, 23]. The labeling process is very simple and there is no need to use genetically modified EVs. This simple imaging technique revealed the spatiotemporal location of systemically injected exogenous EVs in target tumors [18]. However, optical imaging is limited to exogenous EVs and fluorescent dyes persist in tissues even after the EVs are degraded. This is because lipid labeling is not specific for intact EVs and fluorescence might remain in degraded EVs [16].

To visualize endogenous EVs and track cell-to-cell communication directly, reporter imaging methods were introduced. Protein markers of EVs, such as CD63, were used to design reporter conjugated to fluorescent proteins [6, 12]. Although surface proteins labeled with fluorescent proteins are widely expressed in EVs, only a subpopulation of EVs is labeled and the signal intensity depends on the amount of reporter protein expression [7, 15]. Alternatively, schemes such as labeling transmembrane domains with a biotin–streptavidin system [16] or using a reporter fused with a palmitoylation signal [15] were also attempted to track endogenous EVs. Reporter fluorescence imaging systems are more specific to EVs than lipophilic dyes. However, they require genetically engineered cells, and whole-body optical imaging of systemically administered EVs is difficult because of the low yield of fluorescence-labeled EVs.

### Bioluminescence reporter system

The bioluminescence reporters are able to unravel the in vivo behavior of EVs with very high sensitivity. Genetically engineered bioluminescent proteins (e.g., Gaussia luciferase combined with transmembrane domains such as lactadherin) could reveal the spatiotemporal distribution of EVs in a quantitative manner [16, 24] in small animals without background autofluorescence. Bioluminescence imaging overcomes the problem of retained or recirculating fluorescence signals coming from retained fluorescence dyes after EV degradation. However, this system has the shortcoming that the luminescence signal is attenuated when its location is deep in the internal organs. Furthermore, similar to fluorescence reporter imaging, the bioluminescent signal depends on the reporter protein expression [7, 15]. These labeling procedures are complicated compared with those of fluorescence dyes, which limits the study of the in vivo distribution and fate of EVs in various cells under different conditions.

### Radionuclide and magnetic resonance imaging of extracellular vesicles

Optical imaging of EVs (either fluorescence or bioluminescence) has an intrinsic limitation of signal attenuation even in small animals, and other noninvasive imaging methods are necessary for clinical applications. Radionuclide labeling of EVs is one of the options. Similarly to bioluminescence imaging, a streptavidin reporter combined with transmembrane domains expressed in EVs was conjugated with ^125^I-labeled norbiotinamide [25]. Another direct radiolabeling method was also tried using ^111^In-oxine, which appeared quite similar to fluorescence dye labeling [26]. Although the whereabouts of fluorescent dyes released from degraded EV membranes are unknown, ^111^In—if freed from the cells (and thus probably from the EVs)—accumulates in the reticuloendothelial system (RES) of the liver, spleen, and bone marrow [27]. These methods were used only to evaluate ex vivo biodistribution, but more recently ^99m^Tc-HMPAO-labeled EVs were also tested to obtain whole-body images using single photon emission computed tomography (SPECT) [28]. The serial imaging of ^99m^Tc-HMPAO-labeled EVs enabled us to understand in vivo kinetics of EVs quantitatively.

Another approach is to use superparamagnetic iron oxide nanoparticles for magnetic resonance imaging (MRI) [29, 30]. Electroporation of exosomes with superparamagnetic iron oxide nanoparticles produced nanoparticle-laden exosomes [30]. The disadvantage of this method is also difficult—a very large amount of EVs should be used because the signal-to-noise ratio is proportional to the amount of particles on MRI and quantification. Although these noninvasive imaging techniques including SPECT, positron emission tomography (PET), and MRI can show EVs even in deep organs, for clinical translation they require relatively expansive facilities and—except for MRI—may have concerns of radiation exposure.

## In vivo fate, distribution, and targeting of extracellular vesicles

### In vivo fate of extracellular vesicles

EVs secreted by various types of cells can be found in the circulation and biological fluids. While a fraction of EVs are degraded by interactions with recipient cells, they are also systemically cleared from the circulation. In vivo clearance studies reveal that EVs are rapidly cleared, so that the half-life is estimated as approximately 2 min [24, 25, 31]. After this rapid clearance from the circulation, EVs were slowly cleared from the liver, spleen, and lungs [24]. Bioluminescence reporters showed shorter half-lives of EVs in most tissues, including the liver, than did fluorescence markers [16]. The rapid clearance of EVs is supposed to be due to the nonspecific interactions of EVs with blood or endothelial cells, given that EVs have been stable in vitro in plasma (i.e., without interactions with cells) and the size of EVs is too large to be permeable to endothelial cells [17, 32].

EVs accumulate in the liver and lungs within 5 min after systemic injection [24, 25]. Investigators interpreted this as the EVs being captured by the RES in the very early phase of circulation. After being retained in the RES, EVs might be degraded. The pattern of rapid clearance is very similar to liposomes [33], which are rapidly cleared by the liver and spleen. Because of the similarity of clearance between liposomes and EVs regardless of origin [26], differences in surface markers of diverse EVs were thought to have little influence, if any, on their systemic clearance/excretion, the authenticity or generality of which is yet to be elucidated.

Reports suggesting rapid RES sequestration of the EVs raised concerns similar to those of liposome reports. In the literature on liposomes, investigators used in-house liposomes and the variability of RES uptake depended on many physicochemical factors such as size, surface charge, and composition [34–36]. The clearance of exogenous EVs mimicked that of liposomes as the proportion of RES uptake of EVs would have been influenced by the purity and composition of EVs. In addition, EV degradation is affected by the optimality of EV production. Both injection of aggregated EVs and immediate aggregation after systemic injection of EVs resulted in pulmonary sequestration [28]. However, unlike liposomes, EV production from various cell sources requires another step of purification. This would have resulted in investigation-to-investigation and batch-to-batch variability. We propose that during the production of EVs every batch should be tested for consistency before further investigating the physiologic roles or the theranostic capability of EVs.

### In vivo distribution and tissue targeting

EVs have specific proteins such as integrins that interact with recipient cells [3], and they were expected to have the capability of in vivo homing and targeting to specific receptors. This property promised application of EVs as therapeutic vehicles for several diseases. For example, EVs derived from mesenchymal stem cells showed therapeutic effects on myocardial or renal injuries by reaching the damaged target tissues [37–39]. However, several studies into therapeutic effects of EVs did not show in vivo distribution of EVs to prove targeting to specific tissues. Thus, imaging-based studies are needed to determine the roles of EVs in targeting especially the remote organs.

Imaging and tracking of EVs could reveal what is happening to EVs in circulation and whether they are targeting specific tissues. Unfortunately, the literature reports are inconsistent depending on the methods of imaging and tracking. For instance, subcutaneously injected melanoma-derived EVs accumulated in the lymph nodes were regarded as a premetastatic niche in one study [11]. Another study using bioluminescence imaging reported that their systemically injected melanoma-derived EVs labeled with ^125^I-biotin accumulated mostly in the lungs and spleen [24]. Fluorescence-labeled exosomes derived from melanoma cells accumulated mainly in the bone marrow and lungs, which was interpreted as an induced metastatic environment [10]. These studies used EVs derived from the same type of cells (melanoma), but showed different targeting effects (lymph nodes, liver, lungs, and bone marrow). As different administration routes and labeling/tracking methods were used, their influence on the in vivo distribution results should be re-examined.

**Fig. 2:**
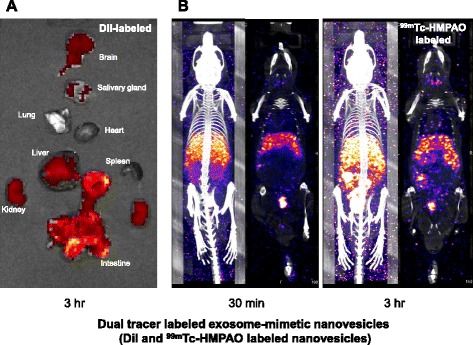
Imaging of dual tracer-labeled exosome-mimetic nanovesicles. Exosome-mimetic nanovesicles [14] were labeled with DiI and ^99m^Tc-HMPAO simultaneously. **a** Ex vivo fluorescence image acquired 3 h after the intravenous injection. Nanovesicles accumulated in the liver, intestine, kidneys, and brain. **b** SPECT images acquired in vivo at 0.5 and 3 h after the intravenous injection. In this specific example, the accumulation pattern was different from fluorescence imaging, showing that the nanovesicles accumulated mainly in the liver and spleen and were few in the intestine. Simultaneous labeling revealed a different distribution which might be partially caused by the differences of behavior of tracers after vesicles degraded in their initially retained tissues

One of the studies revealed that the reason for the different results was the labeling methods. Lipophilic fluorescent dye remained in the tissues even after vesicles were degraded while bioluminescence reporters did not [16]. In our preliminary study, simultaneous DiI and ^99m^Tc-HMPAO labeling showed different distribution patterns (Fig. 2). In vivo distribution was also affected by the protocols of labeling EVs extracted from labeled donor cells or labeling after EV purification [40]. EVs labeled with fluorescence dye showed higher accumulation in the liver, while fluorescence-labeled EVs collected from fluorescence-labeled donor cells showed more specific accumulation in the injured tissue and less in the liver. These results imply that both free forms and metabolites of dyes or tracers from degraded EVs have to be considered in the interpretation. Because each labeling method has its advantages and limitations, a multimodal approach is encouraged [16]. Multimodal tracking in the specific organs could also help to understand the in vivo kinetics of EVs.

There is a concern that lung and liver uptake is sometimes interpreted as RES uptake of circulating EVs. RES accumulation of labeled white blood cells has been well known. According to textbook knowledge [27], systemically administered autologous white blood cells first migrate to the lungs and stay there for a short while, and then move to the inflammation sites or RES tissues including the bone marrow. Immediate lung uptake of EVs, liposomes, or inorganic nanomaterials is considered to be due to the aggregation of these materials, which is hardly a physiologic phenomenon [34, 41]. When preparation of radiolabeling was suboptimal, EVs also accumulated in the lungs [28]. On the other hand, liver uptake of EVs could be due to hepatocellular uptake of EVs rather than uptake by the RES and formation of metabolites may also result in hepatobiliary excretion and gastrointestinal tract accumulation. A recent study showed that the gastrointestinal activity indicated hepatobiliary excretion [42], which varied according to the routes of administration, cell sources, and most notably the time after administration.

Although in vivo distribution of EVs including organ-specific accumulation is closely related to systemic functions, biological effects of EVs in target tissues should be proven as mediators of intercellular communication by imaging studies. Microscopic imaging studies revealed that the exchanges of biomaterials mediated by EVs at the cellular level eventually showed phenotypic changes. For instance, benign tumor cells, which took up EVs secreted by malignant cancer cells, changed their phenotypes, which were directly visualized by the Cre-LoxP system with green fluorescent protein [43, 44]. In the future, to clarify the physiology of EVs, imaging of EVs should focus on molecular changes in target cells as well as in vivo distribution and tissue targeting of EVs.

### Cell type-specific in vivo distribution

The in vivo distribution of EVs depended partly on their cellular origin. Although distribution study results were affected by the labeling methods as already described, cell type specificity, if any, is also important to understand whether EVs have a ‘homing’ capability to recipient cells. A study of biodistribution as a function of cell sources was performed using fluorescence dye-labeled EVs [42]. The size of EVs derived from different sources (muscle, melanoma, and immature bone marrow) was similar (around 100 nm) and they accumulated mostly in the liver and in the spleen. The uptake and clearance pattern was almost similar despite their different origin and only the amount of uptake was slightly different. Another study using both fluorescence dye and ^111^In-oxine labeling detected that EVs of different cell origins had similar distribution patterns, which also showed accumulation mainly in the RES. Liposomes with similar size and exosome-mimicking liposomes (liposomes synthesized from lipid extracts of exosomes) showed similar distribution in this report [26].

Studies of tumor targeting or inflammatory tissue targeting by EVs produced inconsistent results. Tumor characteristics or inflammation could affect the in vivo distribution by influencing the homing behavior of EVs. Exosomal membrane fused with specific integrin receptor ligand such as RGD peptide showed a possibility of tumor-specific accumulation of EVs using fluorescence imaging [45]. The surface-modified EVs were accumulated more in the tumor than in the liver or spleen. Ohno et al. used fluorescence-labeled EVs to target epidermal growth factor receptor-positive breast cancer cells and showed tumor accumulation, although the degree of accumulation was much less than in the liver and spleen [18]. Another study using exosome-mimetic nanovesicles derived from macrophages without surface modification indicated that they were distributed prominently in the tumor [14]. However, another study found that tumor accumulation of EVs was minimal just like liposomes [26]. Approximately 3 % of EVs derived from human embryonic kidney cell lines accumulated in the tumor tissues nonspecifically, which could be due to enhanced permeation and retention of EVs [42]. Mesenchymal stem cell-derived EVs tended to accumulate in the injured tissues, although they also accumulated in the liver and spleen [40]. The underlying mechanism of tumor accumulation of EVs remains unknown. A number of in vivo studies did not compare the distribution of EVs with that of liposomes as controls. Because the cellular uptake of EVs is greater than that of liposomes in vitro in specific cells, and uptake is dependent on the recipient cell types [46], a comparison with liposomes of similar size will be needed to elucidate the active targeting capabilities of EVs.

To sum up, EVs of similar sizes were cleared by the RES and mainly accumulated in the liver, spleen, and lungs, while protein and lipid components that vary by cellular origin have a minor effect to change the gross accumulation and clearance patterns of EVs from the target tissues. Although tumor or inflammatory tissues may affect the accumulation pattern, in most studies the uptake of EVs by the liver and spleen is attributed to RES clearance. It is not directly evident whether accumulation of EVs in tumor or injured tissues is mediated by active targeting or enhanced permeation and retention, or both. For now, the in vivo EV distribution reports imply that the intercellular communication mediated by EVs mostly takes place between neighboring cells rather than the donor cells and the distant targets, which might be limited by RES clearance and/or hepatocellular excretion.

### Extracellular vesicles for brain delivery

Among the issues of in vivo distribution, it is unique and important to understand whether extraneous EVs are transferred to the brain. To use EVs as possible therapeutics for brain disorders, understanding the in vivo distribution of brain-targeted EVs is imperative. In general, delivery of nano-sized vesicles to the brain has been considered to be restricted because of the blood–brain barrier (BBB). Systemically injected luciferase-labeled EVs were minimally found in the brain tissue regardless of cell types [16, 24]. Radionuclide-labeled EVs indicated almost no accumulation in the brain tissue when EVs were systemically administered [25]. As mentioned previously, most extraneous EVs are captured in the liver and spleen or in the lungs [24], which could also hamper the targeted delivery to the brain as well as to other organs or target tissues. These findings suggest, so far, that the intercellular communication using EVs across the BBB might hardly take place.

Nevertheless, therapeutic application has been attempted in small animals using enhanced brain delivery of EVs. Alvarez-Erviti et al. [20] engineered EVs to carry rabies viral glycoprotein (RVG) and showed their therapeutic potential as a small interfering RNA (siRNA) transporter to cross the BBB to treat Alzheimer’s disease in mice. RVG modification of EVs enhanced brain accumulation about twofold compared with nonmodified EVs [42], although brain accumulation was much less (1–2 %) than in the liver and spleen (70–80 %).

Intranasal delivery of EVs, another promising administration route to the brain, was also tried [47, 48]. Direct delivery of stem cells via the nasal route resulted in cells spilling to the lungs, which later caused tumors [49]. Intranasal delivery of inorganic nanoparticles or peptides/antibodies was inefficient [50, 51]. Delivery of nucleic acids is not easy because they are unstable in the extracellular milieu, and thus exosomal packaging of therapeutic RNAs or peptides might prove better. In the future, just like other nanovesicles [50], engineered EVs to target the brain via the best route of brain delivery, such as the nasal route, should promote therapeutic applications of EVs.

## Conclusion

Imaging of EVs is essential to understand the physiology of EVs and to apply EVs as therapeutics for various diseases. The simple and commonly used tracking is carried out with lipophilic labeling of EVs either using fluorescent dyes or radiolabeled dyes. However, accurate tracking of EVs was limited due to nonspecificity of labeling and retention or recirculation of labels after degradation. Furthermore, optical imaging has the issues of limited penetration depth and potential toxicity of substrates in the case of luciferin. In the future, for clinical application of EVs, radionuclide imaging and MRI may be used as noninvasive imaging methods without these disadvantages.

Even though distinctive roles of EVs for intercellular communication are mediated by the complex and specific composition of EV lipids and proteins, systemic distribution and clearance did not yet disclose the differences according to the EV origins and compositions. In vivo distribution of EVs appears to be similar to artificial nanovesicles such as liposomes. EVs are rapidly cleared by the RES or excreted via the liver or kidneys, which may limit their reach to certain target tissues; surface modification to reduce nonspecific uptake may also be required for eventual clinical application of EVs as therapeutics.

Studies of the secretion of EVs by various cells under diverse conditions suggest that there are many subsets of EVs composed of different genetic materials and proteins including surface markers and other biomaterials [52, 53]. If we wish to make a library of EVs of interest, including their roles in physiology and their future therapeutic potential, information regarding their in vivo distribution, clearance, and kinetics should be noted for each subtype of EVs. To elucidate the physiology of various subsets of EVs, novel methods of isolation and purification of these subsets as well as efficient methods for their in vivo characterization will be needed to understand intercellular communication between EV donor cells and receptor cells or distant organs.

## Abbreviations

BBB: blood–brain barrier
EV: extracellular vesicle
MRI: magnetic resonance imaging
RES: reticuloendothelial system
RVG: rabies viral glycoprotein
siRNA: small interfering RNA
SPECT: single photon emission computed tomography
